# Goblet Cell Adenocarcinoma of the Appendix: A Systematic Review and Incidence and Survival of 1,225 Cases From an English Cancer Registry

**DOI:** 10.3389/fonc.2022.915028

**Published:** 2022-07-12

**Authors:** Kieran Palmer, Scott Weerasuriya, Kandiah Chandrakumaran, Brian Rous, Benjamin E. White, Sangeeta Paisey, Rajaventhan Srirajaskanthan, John K. Ramage

**Affiliations:** ^1^ Barts Cancer Centre, St Bartholomew’s Hospital, London, United Kingdom; ^2^ Department of Critical Care Medicine, King’s College Hospital National Health Service (NHS) Foundation Trust, London, United Kingdom; ^3^ Surgical Division, Hampshire Hospitals National Health Service (NHS) Foundation Trust, Basingstoke, United Kingdom; ^4^ National Health Service (NHS) Digital, Leeds, United Kingdom; ^5^ Faculty of Life Sciences and Medicine, King’s College London, London, United Kingdom

**Keywords:** goblet cell adenocarcinoma, goblet cell carcinoid, goblet cell carcinoma, appendix tumour, mucin-secreting tumour

## Abstract

**Background:**

Goblet cell adenocarcinoma (GCA) of the appendix is a rare and aggressive tumour with varying nomenclature and classification systems. This has led to heterogeneity in published data, and there is a lack of consensus on incidence, survival, and management.

**Methods:**

We provide an overview of GCA with a comprehensive systematic review using Preferred Reporting Items for Systematic Reviews and Meta-Analyses (PRISMA) methodology and a retrospective analysis of all cases recorded in the English National Cancer Registration and Analysis Service database between 1995 and 2018. The Kaplan–Meier estimator was used to calculate overall survival, and Cox proportional hazards regression was used to identify prognostic factors.

**Results:**

The systematic review demonstrated an incidence of 0.05–0.3 per 100,000 per year among North American registry studies. The 1-, 3-, and 5-year survival rate was 95.5%, 85.9%–87.6%, and 76.0%–80.6%, respectively. Age, stage, and grade were identified as prognostic factors for survival. Our analysis included 1,225 cases. Age-standardised incidence was 0.0335 per year in 1995 and gradually rose to 0.158 per year in 2018. The 1-, 3-, and 5-year survival rate was 90.0% [95% confidence interval (95% CI): 85.4–94.0], 76.0% (95% CI: 73.8–80.9), and 68.6% (95% CI: 65.9–72.2), respectively. On univariate Cox regression analyses, female sex, stage, and grade were associated with worse overall survival. On multivariate analysis, only stage remained a statistically significant prognostic factor.

**Conclusions:**

GCA of the appendix is rare, but incidence is increasing. We report a lower incidence and survival than North American registry studies. Higher stage was associated with decreased survival. Further prospective studies are required to establish optimal management.

## Introduction

Goblet cell adenocarcinoma (GCA) of the appendix is a rare mucus-secreting tumour that can exhibit both mucinous and neuroendocrine differentiation (1). Depending on the grade and the depth of invasion, GCA can also demonstrate a varied disease course (2). This can range from benign and slow growing to aggressive with significant malignant potential (2). These characteristics have led to considerable variation in nomenclature over time, with GCA having been previously termed adenocarcinoid, mucinous carcinoid, composite composite goblet cell carcinoid (GCC)-adenocarcinoma, adenocarcinoma ex-goblet carcinoid, crypt cell carcinoma, and more recently goblet cell carcinoma or goblet cell carcinoid. There has however been a recent movement away from the term carcinoid, with GCA being preferred. This is because GCA displays inconsistent immunohistochemical staining for neuroendocrine markers and is only rarely associated with hormone hypersecretion syndromes. It has also become apparent that GCA is more aggressive than stage-matched appendiceal neuroendocrine neoplasms (NENs) (3, 4).

Due to its rarity, the exact incidence and survival of GCA have been difficult to ascertain. Most existing published data are derived from registry studies, which are limited by the changes in nomenclature over time, causing inconsistencies in diagnosis and reporting. The remainder of the literature is composed of small retrospective cohort studies and case series, often from single institutions. Many review articles have been published; however, their conclusions are rarely specific to GCA, as they tend to include numerous other types of appendiceal neoplasms. The existence of various conflicting grading and staging systems further complicates classification, although there is an emerging consensus on the latter. In the eighth edition of the Union for International Cancer Control staging manual, it is stated that GCA should be staged similarly to an adenocarcinoma, where “T” category is defined by the depth of invasion rather than the size as is the case in appendiceal NEN (5).

The management of GCA comprises a surgical strategy of a right hemicolectomy for any stage of localised disease, with possibly a prophylactic bilateral salpingo-oophorectomy in women due to the high risk of gynaecological metastases. For more widespread disease, systemic chemotherapy using a 5-fluorouracil (5-FU)-based combination regimen is commonly used. Cytoreductive surgery with heated intraperitoneal chemotherapy (CRS-HIPEC) has been used in patients with peritoneal spread. There are very limited prospective data and no phase III trial data to support these treatment recommendations, so their clinical utility remains uncertain.

Given the rarity of GCA and previous inconsistencies in terminology, grading, staging, and clinical management, we set out to provide an up-to-date overview of GCA. We aimed to perform a systematic review of the literature and to present the largest series of registry data from England to date, with age-standardised incidence and survival data.

## Methods

### Systematic Review

A systematic review was conducted in accordance with the Preferred Reporting Items for Systematic Reviews and Meta-Analyses (PRISMA) guidelines. The online databases Medical Literature Analysis and Retrieval System Online (MEDLINE), Excerpta Medica Database (EMBASE), and PubMed were searched on 18th February 2022, using the following free-text terms and Medical Subject Headings (MeSH): (“goblet cell tumor*”) OR (“goblet cell tumour*”) OR (“goblet cell carcinoma*”) OR (“goblet cell neoplasm*”) OR (“goblet cell carcinoid”) OR (“goblet cell adenocarcinoma*”) OR (“adenocarcinoid) OR GOBLET CELL”/OR “GOBLET CELLS”/OR NEOPLASM/OR “CARCINOID TUMOR”/AND (“appendix”) OR (“appendiceal”) OR “APPENDIX CANCER”/OR “APPENDIX TUMOR”/OR “APPENDIX CARCINOMA”/AND APPENDIX/AND “APPENDICEAL NEOPLASMS”/. Additional relevant papers were sourced via a grey literature search, a Google Scholar search, and a review of the reference lists of selected articles.

Following the removal of duplicate articles, 471 papers progressed to screening. Articles not in English, conference papers, commentaries, broad literature review articles, and animal studies were excluded, as were case reports of only one or two cases, as these were felt not to sufficiently contribute to the literature in terms of incidence or survival. A total of 124 full-text articles were assessed for eligibility. Articles including information on incidence, prevalence, and survival were included as were other clinically relevant publications. Twenty articles were excluded. Seven articles were excluded, as they only included one or two cases of GCA. One abstract and one letter to the editor were also excluded. Two consensus guidelines were excluded, as they provided no new data or statistical analyses. One paper was excluded, as it did not include GCA. Six papers were excluded, as they provided no subgroup analysis for GCA of the appendix. One paper was excluded, as it involved mixed pathology. One review was excluded due to lack of relevance. Two authors (KP and SW) independently reviewed each paper prior to acceptance, and the results were reviewed by JR.

### Registry Analysis

This was a retrospective study of prospectively collected data of tumours in England recorded in the National Cancer Registration and Analysis Service (NCRAS) as Appendix GCA between 1st January, 1995, and 31st December 2018. The pathology of all tumours treated within the NHS (98%–99%) is required to be registered in NCRAS. Some private institutions submit data to NCRAS, but this is incomplete (6). Monthly central returns are made from all hospitals using Cancer Outcomes and Services Dataset (COSD). NHS Digital requests copies and registration of all pathology reports. Dates of death are obtained from the Office of National Statistics and linked to the data.

In addition to analysing all cases diagnosed between 1995 and 2018, a subgroup analysis was also performed for cases occurring after 2009. It was felt that the data after this date were more likely to be accurate following the 2008 Tang et al. (7) publication, which presented a new grading system and advised staging GCA similarly to appendiceal adenocarcinoma.

The age-standardised incidence was calculated per 100,000 patients per year. Five age groups were created based on clinical reasoning after consultation with JR and RS. Tumours were grouped into stages 1–4. Where right hemicolectomy was performed, the staging data included the updated findings from completion surgery. Categorical variables were outlined in frequencies and percentages. Continuous variables were provided as median and interquartile range (IQR). Pearson’s chi-square test was performed to evaluate the difference between groups and the significance. The primary end point was overall survival (OS). This was selected over disease-specific survival (DSS) due to the lack of comorbidity and cause of death data. The Kaplan–Meier-predicted OS was calculated up to the date of death or date of the last follow-up (censored) and given with 95% confidence interval (95% CI). The Mantel–Cox log rank test was used to evaluate statistical differences in survival between groups. The hazard ratio (HR) was estimated with Cox proportional hazards regression model. A *p* value <0.05 was deemed statistically significant. Statistical analyses and graphical plots, including Kaplan–Meier curves, were done using Stata 17 (Stata Corp. LLC, Texas).

During the period studied, there were 1,354 GCA tumours, of which 1,225 (90.5%) were confirmed GCA of the appendix. The other 129 were non-appendiceal GCA and were excluded.

## Results

### Systematic Review

**Figure 1:**
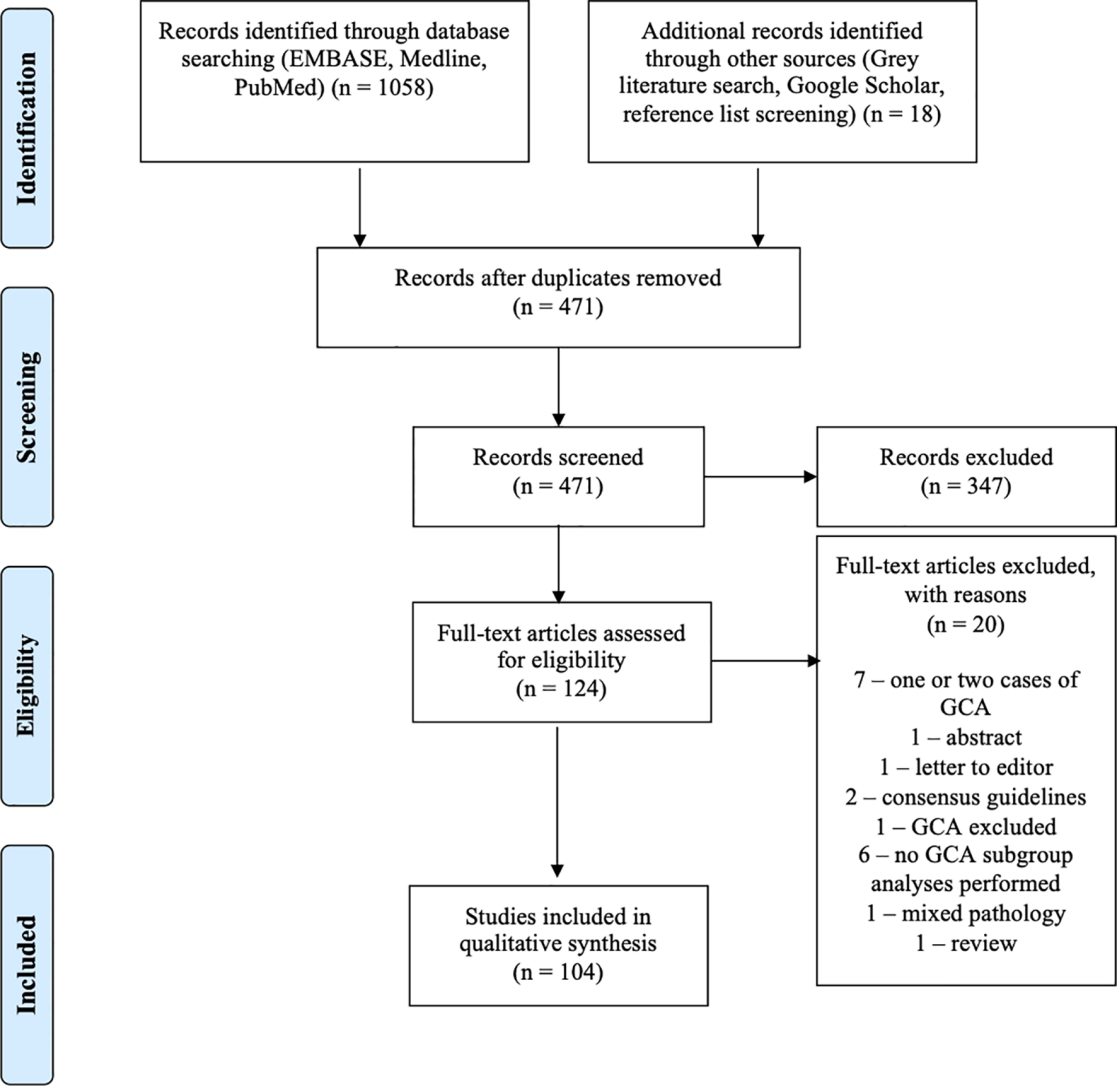
Preferred Reporting Items for Systematic Reviews and Meta-Analyses (PRISMA) flowchart.

Our review included 104 studies (**Figure 1**). These consisted of one meta-analysis (8), one systematic review (9), one prospective cohort study (10), 18 registry studies (2, 3, 11–26), 66 single or multicentre retrospective analyses (4, 7, 27–90), four inter-user variability studies (91–94), and 13 case series (95–107). A quantitative meta-analysis of the studies was precluded by significant heterogeneity in the nomenclature and data. The largest study to date was published by Fields et al. (18) in 2019 and documents 2,552 cases of GCA from the National Cancer Database (NCDB).

#### Epidemiology

The incidence of GCA of the appendix was described in four studies (3, 17, 26, 89). From three reviews of the Surveillance, Epidemiology and End Results (SEER) registry and one of the British Columbia Cancer Agency (BCCA) database, the documented incidence per 100,000 varied between 0.05–0.3 per year (3, 17, 26, 89). This is higher than a previous analysis of all primary neoplasms of the appendix from the SEER registry, which reported an annual incidence of 0.012 per year, of which GCA made up 13.8% (25).

The incidence of GCA is increasing (3, 14, 16, 17, 58, 89). In an analysis of the SEER registry between 1973 and 2014, 98.3% of cases were diagnosed between 1994 and 2014 (17). The rate of rise is increasing in recent years, with another SEER database study finding that only 37.8% of GCA were diagnosed between 2004 and 2009, with the remaining 62.2% diagnosed between 2010 and 2016 (16). A similar trend was noted in the NCDB analysis, in which 96% of the cases of GCA were seen post-2010 (14). Other studies note a rising incidence of all NENs, which may be attributable to an increased awareness and coding of these tumours, an increased rate of detection, other factors, or a real rise (108). The proportion of GCA among all appendiceal neoplasms varied between 10% and 23% in the larger registry studies (11, 12, 17, 18, 21, 23, 25, 26) likely due to inconsistencies in both the inclusion of benign appendiceal neoplasms and GCA nomenclature.

The mean and median age at diagnosis were most commonly reported between 50 and 60 years (12, 14–18) and was 57 years in the largest series (18). Only one large registry study reported a median age out of this range at 43 years (23). GCA is more common in Caucasian patients, who account for 80-90% cases (3, 11, 12, 14–20, 25, 26). There was no sex preponderance in most studies (3, 11, 12, 14, 16, 18, 20, 23, 25, 26); however, this was variable in the smaller retrospective studies and case series. Infection with schistosomiasis was the only potential environmental risk factor identified, although it should be noted that only three cases of combined appendiceal schistosomiasis and GCA were included in this study (63).

The presence of synchronous or metachronous secondary malignancy with GCA has previously been described (103) and was seen in 10% of cases in one analysis of the SEER registry, with a reported estimated standardised incidence ratio of 1.55 (95% CI: 1.23–1.92) (21). This was greater than for appendiceal adenocarcinoma and malignant carcinoid (21).

#### Clinical Presentation

The most common presenting feature of GCA is acute appendicitis (34, 39, 40, 42, 54, 56, 66, 68, 72, 77, 97, 99, 102), followed by non-specific abdominal pain or an abdominal mass (4, 28, 44, 52, 58, 62, 65, 71, 73, 80, 81, 98). Appendicitis is common in low-grade and localised disease (7, 52, 55, 89) and non-specific abdominal pain with or without abdominal mass in higher-grade or metastatic disease (7). Appendiceal perforation was reported by multiple authors (9, 10, 29, 40, 52, 55, 56, 62, 64, 75, 78, 102, 103) and was observed in 23% of cases in one systematic review (9). While some series documented higher rates of perforation (29, 56, 75, 78), this may be a result of publication bias in smaller studies. Appendiceal perforation is more common in lower-grade and localised disease (40, 55). Hormonal hypersecretion syndromes, including carcinoid syndrome, are uncommon in GCA, with only a handful of cases in the literature (7, 58, 74).

Tumour location within the appendix (base compared with apex) was not well documented and was inconsistent among reporting studies (4, 28, 34, 39, 62, 81, 102, 105). Tumour size ranged from 1 to 250 mm (2, 4, 14, 18, 28, 43, 56, 75, 102, 103); however, there was a discrepancy in the method of tumour measurement, with some authors using the maximum tumour diameter and others using length of tumour extension.

The description of staging varied between studies. Authors rarely specified whether staging was based upon index or completion surgery. In larger registry studies using the TNM system, most tumours presented with stage II disease (14, 18–20, 28, 39, 52, 58); T3 tumours were found in 49%–60% and N0 in 81%–87% of cases (11, 12, 23). In studies classifying GCA as local, regional, or metastatic disease, 51%–64% of cases were described as local (3, 16, 17, 26). While metastases were found in only a relatively small proportion of cases in the registry studies (7%–18.7%) (3, 11, 12, 16–20, 23, 26), stage IV disease was more common in retrospective studies and case series (7, 37, 39, 44, 49, 52, 58, 73). It is likely that this finding is the result of referral bias, as higher rates of metastatic disease were reported by tertiary centres, with lower rates reported by district general hospitals and national registry studies (52). The most common sites of metastases were the peritoneum, liver, small bowel, and ovaries (11, 28, 37, 49, 52, 89, 90).

#### Diagnostics and Surveillance

Similar to appendiceal NEN, the diagnosis of GCA is usually made incidentally on postoperative histology. Diagnostic workup comprises postoperative staging with cross-sectional imaging. Follow-up involves surveillance CT scanning to monitor for recurrence.

Preoperative CT findings are variable in GCA, and there are no characteristic radiological features. This makes diagnosis prior to histological assessment challenging (62). There is however a possible correlation between preoperative CT results and subsequent tumour grade. In a retrospective study of 27 patients, a CT result describing typical appendicitis was more commonly associated with low-grade GCC group A as per the Tang grading system (7), while description of a “mass” or “prominent appendix without peri-appendiceal infiltration” corresponded better with signet ring cell adenocarcinoma goblet cell carcinoid group B (62).

Functional imaging including octreotide scanning, Iodine 123 metaiodobenzylguanidine, and Ga-DOTATATE PET is mainly negative in GCA (28, 39, 52, 54, 58, 102). Fluorodeoxyglucose positron emission tomography (FDG-PET) may have better sensitivity (39, 54); however, the evidence for this is limited. Chromogranin A and B is rarely raised (39, 102). Elevated serum carcinoembryonic antigen (CEA), Carbohydrate antigen 19-9, and CA-125 were reported in some studies (12, 39, 52, 54, 74, 102), although there is a paucity of prospective data to demonstrate their clinical utility in monitoring for recurrence. Given the increased incidence of synchronous or metachronous colonic malignancy (103), some authors suggest performing follow-up colonoscopies. This is endorsed in the European Neuroendocrine Tumor Society guidelines (109, 110); however, the optimal frequency of endoscopic surveillance has not been determined.

#### Pathology, Immunohistochemistry, and Genetics

GCA develops from pluripotent intestinal crypt base stem cells, which show mucinous and neuroendocrine differentiation. The defining histological feature is the focal presence of goblet-shaped epithelial cells with intracytoplasmic mucin that congregates in the lamina propria of the submucosa (4, 7). GCA stains positive on periodic acid-Schiff (PAS) staining of mucin, which helps to distinguish it from an appendiceal NEN (4). The two tumours can be further differentiated by their proliferative indices, as measured by Ki-67, which is significantly higher in GCA (4).

With regard to immunohistochemistry, CEA expression appears to be the predominant differentiator, present in GCA but not in appendiceal NEN (68, 100). There was variable expression of the neuroendocrine markers insulinoma-associated protein 1 (INSM1), chromogranin, synaptophysin, and CD56 reported across studies (32, 38, 78, 100).

Genetic studies in GCA were inconclusive on histogenesis. A commonly reported aetiopathogenic factor was the occurrence of Tumour Protein 53 mutation (33, 45, 79), which in one study was only found in poorly differentiated tumours, possibly suggesting that it is the cause of high-grade transformation (45). However, the presence of TP53 mutation was inconsistent between studies (68, 75, 100). Multiple authors reported no Epidermal growth factor receptor, BRAF (59), KRAS (41, 42, 59), or Adenomatous polyposis coli (APC) (41, 42) mutations in GCA, suggesting that its molecular pathogenesis is significantly different from that of colorectal adenocarcinoma, although again this was not a unanimous finding (33, 45). Low rates of microsatellite instability (59), programmed death ligand 1 (PD-L1), and tumour mutational burden (TMB) suggest that GCA is an immunologically “cold” tumour (33).

#### Grading

The grading of GCA has varied over time (7, 40, 55, 82, 90). In 2008, Tang et al. (7) developed a three-tiered grading system (A–C) based upon the degree of cytologic atypia, desmoplasia, and cellular differentiation. Lee et al. (90) subsequently devised a simpler two-tiered system and created a histological scoring system based upon the presence of cytologic atypia, desmoplasia, and solid growth pattern. More recently, with an increasing consensus that GCA should be classified as an adenocarcinoma, Yozu et al. (40) proposed that grading should depend upon the proportion of the tumour that shows tubular or clustered growth pattern, with a value of >75% tubular or clustered growth for low grade (grade 1), 50%–75% for intermediate grade (grade 2), and <50% for high grade (grade 3). The World Health Organisation (WHO) Classification of Tumours 5th Edition, volume 1, supports the reclassification of goblet cell carcinoma as an adenocarcinoma (111). The Ki-67 proliferation index has been used to grade GCA (28); however, unlike in NEN, Ki-67 does not appear to correlate with prognosis (58, 64).

Four inter-user variability studies have found significant discordance in grading among pathologists (91–94). One study that directly compared inter-user agreement between the Lee et al. (90) and Tang et al. (7) classification systems found that while gastrointestinal specialist pathologists had substantial agreement for both two- and three-tiered systems, non-gastrointestinal-trained pathologists had significantly better agreement using the two-tiered system, even though their overall agreement was less (93). Subspecialty gastrointestinal pathologist review is therefore recommended in the case of GCA, but ultimately, there is a clear need for an international consensus on a single classification system.

The proportion of each grade at presentation varied depending on which classification system was used. The SEER database and NCDB grade GCA as: “1: well differentiated”; “2: moderately differentiated”; “3: poorly differentiated”; or “4: undifferentiated” (20). However, unknown grade was reported in 55%–89% of cases in most of the analyses of these registries, which prevents any meaningful conclusions on grading being drawn (2, 12, 14, 16, 19, 20, 22, 23). In one SEER analysis of 909 cases of “goblet cell carcinoid” with complete grading data, 48% were classified as grade 1, 24.3% as grade 2, 23.9% as grade 3, and 3.8% as grade 4 (17).

Grade of GCA has been shown to correlate with prognosis. Tang et al. (7) reported the 5-year DSS rate as 100%, 36%, and 0% for group A, B, and C, respectively, with a similar pattern observed in terms of OS in various retrospective analyses (40, 58, 90). Histological grade has been shown to remain an independent prognostic factor when controlled for stage in multicentre studies (40, 90).

#### Management

There is a lack of high-quality randomised controlled trial evidence to support any specific management strategies in GCA. The only prospective study that provided treatment recommendations had a very small sample size (10). In general, management decisions appear predominantly based upon tumour stage and grade. Surgery was performed in more than 98% of cases in two large registry studies (17, 18). Chemotherapy was administered in 14.7%–16.0% of cases, although it was unclear what proportion was in the neoadjuvant, adjuvant, or palliative setting (12, 18). Radiotherapy was very rarely used (22). Targeted treatment, immunotherapy, ablative therapy, and peptide receptor radionucleotide therapy have not systematically been studied.

##### Locoregional Disease

Right hemicolectomy was often performed following index appendicectomy (7, 23, 39, 54, 58, 89, 102, 103). In registry studies, hemicolectomy or more extensive surgery was performed in 42%–87% of cases (3, 12, 16–18, 23, 25). Bilateral salpingo-oopherectomy has been used as a prophylactic surgical strategy in female patients (28, 54, 58) and has been endorsed by ENETS guidelines (109).

Hemicolectomy may confer a survival advantage over appendicectomy alone in stage I–III disease; however, this was not a unanimous finding in the literature (2, 15, 52). In fact, in a retrospective study specifically stratifying by tumour “T” stage, hemicolectomy only conferred a statistically significant survival benefit in T3 and T4 tumours (5-year survival rate 85.4% vs. 82.0%, *p* = 0.028), with no difference in survival seen in T1 and T2 tumours, regardless of appendicectomy or hemicolectomy (83.6% vs. 87.3% *p* = 0.176) (15). Some authors therefore have argued that small (<1 cm), low-grade, and localised tumours with a low proliferation index can be managed with appendicectomy alone (8, 15, 73, 103); however, in reality, this situation is a rare clinical occurrence (109). Negative surgical margins have been associated with improved survival in both appendicectomy and hemicolectomy (5-year OS 83.6% vs. 47.2%, *p* < 0.001) (18), as has harvesting greater than 12 lymph nodes (HR 0.51, 95% CI: 0.34–0.77, *p* = 0.0015) (12).

Recurrence occurs despite high rates of secondary completion surgery. In one multicentre study, 16% of patients radically resected with stage I–III disease had recurrence (89), and even higher rates of 20% and 29% were documented in other retrospective cohort studies (54, 58). Recurrence was significantly higher in node-positive disease (56), Tang class B disease, or patients without appendicitis at presentation (89). The 5-year recurrence-free survival has been estimated at 73.6%–76.0% (28, 89).

The use of adjuvant chemotherapy was investigated in several studies (2, 14, 15, 52, 54, 56, 73, 89). Across localised and regional disease, this was given to 14%–17% of patients (14, 89). It was more commonly used in younger patients (14), men, those with higher grade or stage tumours (14, 15), and those undergoing hemicolectomy (15). In one study of 1,083 stage I–III GCA, adjuvant chemotherapy was associated with improved overall survival (HR 0.28, 95% CI: 0.12–0.54, *p* = 0.002) (15). A consistent survival advantage from adjuvant chemotherapy in lymph node-positive (14, 18) or stage III (2) disease was seen in multiple studies. This effect was not seen in stage II disease (2, 14, 18) or when stage I–III were grouped together in other studies (52, 73, 89).

One study investigated the use of CRS-HIPEC in eight patients with localised disease deemed high risk for peritoneal metastases, as defined by a perforated appendix, a peri-appendicular abscess, or a resection margin <1 mm (10). Four patients received neoadjuvant chemotherapy prior to CRS-HIPEC, and five received adjuvant chemotherapy. The 5-year OS was 100%, with a median follow-up of 3.5 years (10).

##### Metastatic Disease

Metastatic disease carries an unfavourable prognosis, with 1-, 3-, and 5-year OS rates in stage IV disease of 73.0%–85.7% (19, 58), 32.9% (19), and 18.0%–18.9% (18, 26, 58), respectively. There is no clear consensus on the optimal management of such patients, and due to the heterogeneity amongst treatments used, it is not possible to compare subgroup survival rates across studies or to identify prognostic factors.

The most common palliative chemotherapy regimens were similar to those used in colonic adenocarcinoma, either 5-FU-based or a combination of capecitabine and oxaliplatin (7, 28, 37, 52, 54). More rarely, authors used a small-cell lung cancer-based regimen such as carboplatin and etoposide, a NEN regimen such as streptozocin and 5-FU, or an ovarian cancer regimen such as carboplatin and docetaxel (58). Some authors combined systemic chemotherapy with targeted therapy such as bevacizumab (58, 101). In the largest series of 2,552 patients, 70.2% of patients with stage IV disease received some form of chemotherapy; however, this was not associated with improved survival (HR 0.9, 95% CI: 0.49–1.82, *p* = 0.86) (18). There were variable results among smaller studies. In one case series of high-grade GCA, patients treated with palliative folinic acid, fluorouracil, and oxaliplatin (FOLFOX) or irinotecan (FOLFIRI) had a progression-free survival (PFS) of 21.5 months and median OS of 32.9 months (37), yet in a retrospective analysis of 24 patients, the PFS was only 5.3 months (52).

The use of CRS-HIPEC in patients with peritoneal spread was investigated in multiple retrospective studies (10, 27, 29–31, 36, 37, 47, 50, 51, 57, 60, 65, 74). Most showed a median OS between 17 and 45 months (29, 47, 50, 57, 65, 74). Disease-free survival was reported as 13–16 months (51, 60). The only prospective study to date reported a median OS of 3.2 years (10); however, this only included 27 patients. CRS-HIPEC has been associated with significantly improved survival compared to CRS alone (39 vs. 7 months, *p* = 0.001) (47).

In patients with peritoneal metastases who have undergone CRS-HIPEC, lower grade (36, 51), a peritoneal cancer index of 0–20 (29, 60, 74), complete resection (29, 35, 51, 60, 65, 74), and adjuvant chemotherapy (47) have all been associated with longer survival. In a series of 24 patients, OS and PFS was significantly higher in patients with a cytoreductive score of 0 (no evidence of disease after resection) compared to a score of 1 (tumour nodules ≤0.25 cm after resection), and the authors recommend only using a cytoreductive score of 0 as a definition of complete cytoreduction in GCA (35). Neither the administration of neoadjuvant chemotherapy nor the type of perioperative chemotherapy was associated with improved survival (29, 47); however, one study found higher *in vitro* drug sensitivity to docetaxel in GCA than in colonic adenocarcinoma (*p* = 0.05) (65). There were relatively low reported morbidity rates associated with CRS-HIPEC, with grade III or higher morbidity ranging between 13.4% and 30.2% (47, 51). While all of the above suggests CRS-HIPEC may be a promising treatment in the case of advanced GCA with peritoneal metastases, most papers included were retrospective cohort studies, and therefore the results may be subject to selection bias.

#### Survival and Prognosis

While GCA has a worse survival than that in appendiceal NEN, it is better than that in colonic adenocarcinoma, mucinous adenocarcinoma, signet ring cell carcinoma, and mixed adeno-neuroendocrine carcinoma (3, 11, 12, 20). In a study of 944 patients, across all grades and stages, median OS was estimated at 13.8 years (20). Among registry studies, 1-, 3-, 5-, and 10-year OS was estimated at 95.5% (19), 85.9%–87.6% (16, 19), 76%–80.6% (16, 18, 26), and 58.7%–67.1% (16, 18), respectively. In retrospective cohort studies and case series, these were markedly lower at 79%–92% (39, 54, 58), 60%–63% (39, 54), 42%–60% (39, 54, 58), and 38% (58), respectively, likely secondary to higher rates of stage IV disease. The 5-year OS for stages I, II, III, and IV has been estimated at 91.1%–100%, 67.0%–90.5%, 36.0%–57.0%, and 4.2%–18.9%, respectively (18, 40).

Age (18, 40, 52), grade (40, 44, 90), and stage (12, 23, 40, 44, 52, 55) have been identified as independent prognostic factors for survival. Male sex (14), lymph node metastases (14, 15, 18), and positive surgical margins (18) have been associated with decreased survival in stage I–III disease on multivariate analyses. The association between tumour size and prognosis was inconsistent (18, 44). In one study, white ethnicity seemed strongly associated with improved OS (HR 0.44, 95% CI: 0.27–0.71, *p* = 0.0008) (12); however, this finding has not been replicated elsewhere.

### Results: Registry Analysis

A total of 1,225 patients were included in our analysis. The demography and characteristics of the population are presented in **Table 1**. The median age was 60 years (IQR 49–69). There was a greater proportion of women in higher age groups; this was statistically significant (**Table 2**). In this study, 1,114 (90.9%) of patients were of white ethnicity compared to 89% of England’s population (112). There was an even distribution of incidence across the Index of Multiple Deprivation (IMD) (113).

**Table 1:** Demography and characteristics of the patient cohort.

Characteristic	1995–2018	2009–2018
Age band		
Under 29 years	27 (2.2%)	12 (1.6%)
30–54 years	426 (34.8%)	266 (35.4%)
55–64 years	319 (26.0%)	188 (25%)
65–74 years	284 (23.2%)	178 (23.7%)
75+ years	169 (13.8%)	107 (14.2%)
Sex		
Men	607 (49.6%)	360 (47.0%)
Women	618 (50.4%)	391 (52.1%)
Ethnicity		
Asian	15 (1.2%)	12 (1.6%)
Black	10 (0.8%)	9 (1.2%)
Mixed race	5 (0.4%)	3 (0.4%)
Other	9 (0.7%)	7 (0.9%)
White	1114 (90.9%)	695 (92.2%)
Unknown	72 (5.9%)	25 (3.3%)
Index of Multiple Deprivation (IMD)		
1 – Least deprived	237 (19.4%)	155 (20.6%)
2	265 (21.6%)	165 (22.0%)
3	267 (21.8%)	155 (20.6%)
4	227 (18.5%)	139 (18.5%)
5 – Most deprived	229 (18.7%)	137 (18.2%)
Grade		
1	89 (7.3%)	77 (10.3%)
2	117 (9.6%)	107 (14.2%)
3	189 (15.4%)	168 (22.4%)
Unclassified	830 (67.8%)	399 (53.1%)
Stage		
1	107 (8.7%)	102 (13.6%)
2	274 (22.4%	261 (34.8%)
3	87 (7.1%)	82 (10.9%)
4	89 (7.3%)	79 (10.5%)
Unclassified	668 (54.5%)	227 (30.2%)

**Table 2:** Age bands by sex.

Factor	1995–2018			2009–2018		
	Men	Women	*p* value	Men	Women	*p* value
Age Band						
Under 29 years	12 (2.0%)	15 (2.4%)	0.001	4 (1.1%)	8 (2.1%)	0.001
30–54 years	249 (41.0%)	177 (28.6%)		154 (42.8%)	112 (28.6%)	
55–64 years	154 (25.4%)	165 (26.7%)		89 (24.7%)	99 (25.3%)	
65–74 years	125 (20.6%)	159 (25.7%)		71 (19.7%)	107 (27.4%)	
75+ years	67 (11.0%)	102 (16.5%)		42 (11.7%)	65 (16.6%)	
*Grade						
1	41 (22.0%)	48 (22.9%)		37 (22.8%)	40 (21.7%)	
2	57 (30.7%)	60 (28.7%)	0.419	49 (30.2%)	58 (30.1%)	0.869
3	88 (47.3%)	101 (48.3%)		76 (46.9%)	95 (48.2%)	
*Stage						
1	45 (16.6%)	62 (21.7%)		43 (16.8%)	59 (21.9%)	
2	151 (55.7%)	123 (43.0%)	0.030	144 (56.5%)	117 (43.5%)	0.013
3	41 (15.1%)	46 (16.1%)		39 (15.3%)	43 (16.0%)	
4	34 (12.5%)	55 (19.2%)		29 (11.4%)	50 (18.6%)	

* Unclassified excluded.

**Figure 2:**
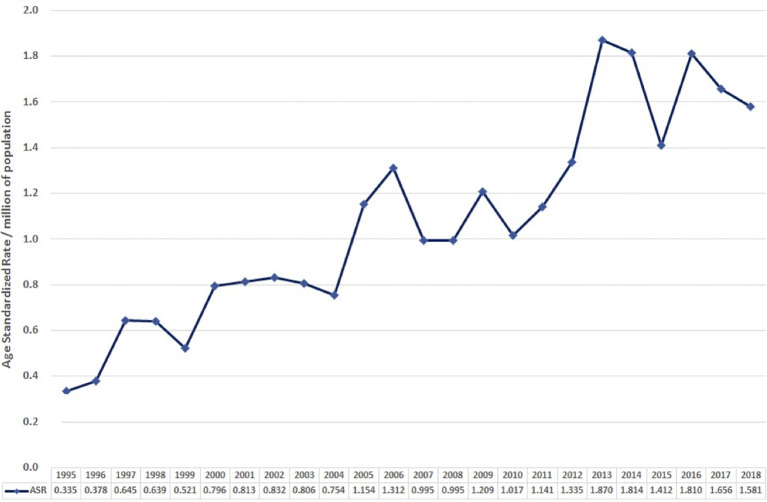
Age-standardised incidence rate (ASR) over time (1995–2018).

The age-standardised incidence in 1995 was 0.0335 per 100,000 per year. Overall, this gradually increased to 0.158 in 2018 (**Figure 2**).

The 1-, 3-, and 5-year survival rate was 90.0% (95% CI: 85.4–94.0), 76.0% (95% CI: 73.8–80.9), and 68.6% (95% CI: 65.9–72.2), respectively. Female sex (*p* = 0.006), higher grade (*p* = 0.001), and higher stage (*p* = 0.001) were significantly associated with lower OS (**Table 3A**). Ethnicity and IMD were not associated with OS. On univariate Cox regression analyses, female sex (HR 1.23, 95% CI: 1.07–1.53 *p* = 0.006), grade 3 tumours (HR 2.85, 95% CI: 1.76–4.61, *p* = 0.001), and stage III (HR 3.34, 95% CI: 1.85–6.02) or stage IV (HR 12.30, 95% CI: 7.14–21.15, *p* = 0.001) disease were significant predictors of worse OS (**Table 4A**). On multivariate Cox regression analysis, only stage (HR 2.90, 95% CI: 2.27–3.71, *p* = 0.001) remained a statistically significant prognostic factor (**Table 4B**).

**Table 3A:** Overall survival by prognostic factors in 1995–2018.

Factor	1-year (95% CI)	3-year (95% CI)	5-year (95% CI)	*p* value
1995–2018 cohort	90.0% (85.4–94.0)	76.0% (73.8–80.9)	68.6% (65.9–72.2)	
Sex				0.006
Men	91.1% (86.8–94.7)	80.6% (77.5–83.9)	73.1% (69.7–76.9)	
Women	89.1% (85.3–92.0)	72.2% (68.7–75.8)	64.2% (60.3–68.2)	
*Grade				0.001
1	95.8% (94.2–97.7)	85.7% (78.4–93.5)	80.1% (71.3–89.0)	
2	95.2% (92.2–97.0)	84.4% (77.5–91.4)	79.1% (70.9–87.2)	
3	83.7% (79.7–85.0)	65.7% (58.5–73.8)	53.3% (45.3–61.5)	
*Stage				0.001
1	94.8% (91.0–97.2)	89.2% (82.9–94 7)	85.7% (78.3–94.3)	
2	96.1% (93.9–98.0)	89.5% (85.4–92.4)	84.5% (81.1–88.4)	
3	88.1% (85.1–90.5)	68.1% (58.0–78.6)	55.8% (44.0–57.6)	
4	60.0% (52.7–67.4)	21.7% (13.1–30.2)	10.0% (3.9–16.9)	

* Unclassified excluded.

Ethnicity and IMD, Index of Multiple Deprivation (IMD): Not significant.

In this study, 751 patients were included in the 2009–2018 subgroup analysis (**Table 1**). Median age was 60 years (IQR 50–70). In addition, 53.1% and 30.2% of cases had their grade and stage unclassified, which were notably less than those of the 1995–2018 cohort. The 1-, 3-, and 5-year survival rate was 90.1% (95% CI: 85.7–94.8), 76.7% (95% CI: 71.8–80.0), and 69.5% (95% CI: 66.1–72.9), respectively (**Table 3B**). In keeping with the initial analysis, female sex (HR 1.34, 95% CI: 1.3–1.74, *p* = 0.029) and higher grade (HR 2.73, 95% CI: 1.60–4.80, *p* = 0.001) and stage (HR 11.70, 95% CI: 6.56–20.86, *p* = 0.001) were associated with decreased OS on univariate analyses (**Table 4A**). On multivariate analysis, only stage remained an independent prognostic factor (HR 2.78, 95% CI: 2.17–3.56, *p* = 0.001) (**Table 4B**).

**Table 3B:** Overall survival by prognostic factors in 2009–2018.

Factor	1-year (95% CI)	3-year (95% CI)	5-year (95% CI)	*p* value
2009–2018 cohort	90.1% (85.7–94.8)	76.7% (71.8–80.0)	69.5% (66.1–72.9)	
Sex				0.028
Men	91.2% (87.1–94.8)	80.5% (75.2–84.0)	73.6% (69.9–77.09)	
Women	89.3% (85.9–92.0)	73.3% (70.0–79.2)	65.8% (60.9–69.4)	
*Grade				0.001
1	96.4% (91.3–98.6)	88.9% (76.4–94.5)	82.2% (73.3–89.1)	
2	95.6% (90.0–98.3)	84.4% (77.5–91.4)	79.1% (70.9–87.2)	
3	84.0% (79.7–90.0)	68.2% (57.3–76.6)	56.4% (49.6–64.5)	
*Stage				0.001
1	94.5% (90.1–97.6)	88.6% (81.3–91 7)	84.8% (76.0–90.3)	
2	96.3% (92.3–98.0)	91.5% (87.1–93.2)	86.9% (82.2–89.8)	
3	88.7% (84.7–93.0)	67.3% (55.1–74.2)	54.1% (43.2–56.6)	
4	59.8% (55.3–64.5)	23.2% (14.2–29.3)	11.3% (4.0–17.3)	

*Unclassified excluded.

Ethnicity and IMD: Not significant.

**Table 4A:** Hazard ratio (Univariate–Cox regression analyses).

Factor	*1995–2018*		*2009–2018*	
	HR (95% CI)	*p* value	HR (95% CI)	*p* value
Sex				
Men	1	0.006	1	0.029
Women	1.23 (1.07*–*1.53)		1.34 (1.3*–*1.74)	
Grade				
1	1		1	
2	1.21 (0.70*–*2.1)	0.510	1.18 (0.61*–*2.26)	0.617
3	2.85 (1.76*–*4.61)	0.001	2.73 (1.60*–*4.80)	0.001
Stage				
1	1		1	
2	0.97 (0.54*–*1.73)	0.917	0.85 (0.45*–*1.60)	0.616
3	3.34 (1.85*–*6.02)	0.001	3.61 (1.95*–*6.75)	0.001
4	12.30 (7.14*–*21.15)	0.001	11.70 (6.56*–*20.86)	0.001

**Table 4B:** Hazard ratio (Multivariate–Cox regression analyses).

Factor	*1995–2018*		*2009–2018*	
	HR (95% CI)	*p* value	HR (95% CI)	*p* value
Sex	1.44 (0.91*–*2.28)	0.124	1.44 (0.89*–*2.30)	0.131
Grade	1.27 (0.94*–*1.72)	0.116	1.34 (0.98*–*1.84)	0.670
Stage	2.90 (2.27*–*3.71)	0.001	2.78 (2.17*–*3.56)	0.001

## Discussion

This study provides a comprehensive overview of GCA of the appendix as a single entity. We present the first and largest registry dataset from England and demonstrate the incidence and survival of a verified population-based cohort presenting multiple institutions over a 24-year period. This, combined with a robust systematic review, provides an extensive account of this rare tumour and its prognosis.

In both our data and the systematic review, the median age at presentation was in the 6th–7th decades, and there was no obvious sex preponderance. Combining both analyses suggests that GCA has an incidence per 100,000 ranging between 0.03 and 0.3 per year and that this is increasing. It is interesting to note that the age-standardised incidence of 0.158 in 2018 seen in our study is less than the most recently documented incidence of 0.3 per year in the analysis by Shaib et al. (3) of the SEER database published in 2016. This may be due to the use of age standardisation in our study. Alternatively, it may be a result of inherent differences between the English and American populations or due to the possibly higher incidental diagnosis rate that comes with the increased patient screening in private healthcare settings. Furthermore, as the SEER database only covers approximately one-third of the US population, analyses of this registry data may not estimate the true population incidence.

While we acknowledge that there is a significant amount of missing staging and grading data in our study, our available data do largely mirror those of previous studies. Patients are most likely to have stage II disease at presentation, and higher grade and stage are associated with worse OS. The 1-, 3-, and 5-year survival rates of 90.0% (95% CI: 85.4–94.0), 76.0% (95% CI: 73.8–80.9), and 68.6% (95% CI: 65.9–72.2) that we observed were however lower than the 95.5% (19), 85.9%–87.6% (16, 19), and 76%–80.6% (16, 18, 26) published in other registry studies. This may be due to unidentified prognostic factors or differences in the treatment modalities used. The effect of presenting grade on survival cannot be inferred due to the missing data in both our study and previous registry studies. Our subgroup analysis for 2009–2018 demonstrated an improvement in the documentation of grading and stage likely because of increased agreement in the classification of GCA in more recent years (5, 7). This more accurate dataset matched the findings of our initial analysis, with female sex, grade, and stage being associated with decreased OS and stage being an independent prognostic factor on multivariate analysis.

In our study, there was a reduced survival in women, which differs to most gastroenteropancreatic NENs, where survival is generally better in women (114). This could be explained by the higher age and stage of the female patients in our cohort; however, the cause of this is unclear. Such a relationship between female sex and worse survival in GCA has not been observed previously, so additional studies are needed to investigate this association.

Due to its rarity and the prior lack of international consensus regarding nomenclature, grading, and staging, the optimal management of GCA remains a challenge. Right hemicolectomy appears to be the most common approach in localised disease and is supported by various international guidelines; however, studies have not shown a statistically significant survival benefit for all stage I–III tumours (15). Adjuvant chemotherapy appears beneficial in lymph node-positive (14, 18) or stage III disease (2). Systemic chemotherapy and CRS-HIPEC have been used in metastatic disease, although there was significant variation in treatment regimens used. In patients with peritoneal metastases, CRS-HIPEC appears to have better outcomes than surgery alone (47). Long-term, prospective, randomised, and phase III trials are required to inform better management protocols; however, due to low incidence, well-powered studies will be challenging.

There are several limitations of this study. Firstly, as discussed, a large proportion of patients within our study did not have their grading or staging classified, which led to their exclusion from the final analyses. This, however, was not dissimilar to previous registry studies in which 55%–89% of tumours had the grade at presentation reported as unknown (2, 12, 14, 16, 19, 20, 22, 23). Additionally, as NCRAS data regarding clinical presentation, diagnostic investigations, or treatment regimens were incomplete or not yet available, the impact of these on survival could not be investigated. Given the advancements in both medical and surgical therapies over the long time period studied, it is possible that treatment strategy could have influenced survival differentially over time. It is interesting to note however that survival was comparable between our total cohort and the 2009–2018 subgroup. The systematic review is limited by the heterogeneity in nomenclature and the variations in grading and staging systems, which potentially introduces inaccuracies when comparing the registry studies. The included cohort studies and case series all contained relatively small patient numbers, with many coming from single institutions. While this reflects the rare nature of GCA, it impairs the generalisability of their findings.

## Conclusion

We have presented age-standardised incidence, survival, and associated prognostic markers of this rare tumour with malignant potential. An improved understanding of GCA among clinicians is needed to achieve optimal patient outcomes. In the future, prospective and appropriately designed randomised trials of this neoplasm are required to inform management protocols.

## Data Availability Statement

The datasets presented in this article are not readily available because source NCRAS data is restricted and cannot be made available. Requests to access the datasets should be directed to kieran.palmer3@nhs.net.

## Ethics Statement

The studies involving human participants were reviewed and approved by Hampshire Hospitals NHS Foundation Trust (IRAS ID: 284875) and received research ethics council approval (REC reference: 20/NW/0342). The ethics committee waived the requirement of written informed consent for participation.

## Author Contributions

Conceptualisation – KP, SW, KC, BR, BEW, SP, RS, JKR. Data curation – KC, BEW, BR. Formal Analysis – KP, SW, KC, BR, BEW, SP, RS, JKR. Funding acquisition – JKR, RS, BEW. Investigation - KP, SW, KC, BR, BEW, SP, RS, JKR. Methodology – KP, SW, KC, BR, BEW, SP, RS, JKR. Supervision – BR, SP, RS, JKR. Writing – original draft – KP, SW, KC, BR, BEW, SP, RS, JKR. Writing – review and editing - KP, SW, KC, BR, B, SP, RS, JKR. All authors contributed to the article and approved the submitted version.

## Funding

Data were extracted from the NCRAS database using a grant from the Neuroendocrine Cancer United Kingdom.

## Conflict of Interest

The authors declare that the research was conducted in the absence of any commercial or financial relationships that could be construed as a potential conflict of interest.

## Publisher’s Note

All claims expressed in this article are solely those of the authors and do not necessarily represent those of their affiliated organizations, or those of the publisher, the editors and the reviewers. Any product that may be evaluated in this article, or claim that may be made by its manufacturer, is not guaranteed or endorsed by the publisher.

## References

[1] Sinno SAJ Jurdi NMH. Goblet Cell Tumors of the Appendix: A Review. Ann Diagn Pathol (2019) 43:151401. doi: 10.1016/j.anndiagpath.2019.151401 31675676

[2] Zakka K Williamson S Jiang R Reid MD Alese OB Shaib WL. Is Adjuvant Chemotherapy Beneficial for Stage II-III Goblet Cell Carcinoid/Goblet Cell Adenocarcinoma of the Appendix? Surg Oncol (2021) 36:120–9. doi: 10.1016/j.suronc.2020.12.003 33360118

[3] Shaib W Krishna K Kim S Goodman M Rock J Chen Z. Appendiceal Neuroendocrine, Goblet and Signet-Ring Cell Tumors: A Spectrum of Diseases With Different Patterns of Presentation and Outcome. Cancer Res Treat (2016) 48(2):596–604. doi: 10.4143/crt.2015.029 PMC4843718 26044156

[4] Jiang Y Long H Wang W Liu H Tang Y Zhang X. Clinicopathological Features and Immunoexpression Profiles of Goblet Cell Carcinoid and Typical Carcinoid of the Appendix. Pathol Oncol Res (2011) 17(1):127–32. doi: 10.1007/s12253-010-9291-5 20640606

[5] Brierley J Gospodarowicz M Wittekind C. TNM Classification of Malignant Tumours. 8th ed. Chichester: Wiley (2016).

[6] Henson KE Elliss-Brookes L Coupland VH Payne E Vernon S Rous B. Data Resource Profile: National Cancer Registration Dataset in England. Int J Epidemiol (2020) 49(1):16–16h. doi: 10.1093/ije/dyz076 31120104 PMC7124503

[7] Tang LH Shia J Soslow RA Dhall D Wong WD O’Reilly E. Pathologic Classification and Clinical Behavior of the Spectrum of Goblet Cell Carcinoid Tumors of the Appendix. Am J Surg Pathol (2008) 32(10):1429–43. doi: 10.1097/PAS.0b013e31817f1816 18685490

[8] Varisco B McAlvin B Dias J Franga D. Adenocarcinoid of the Appendix: Is Right Hemicolectomy Necessary? A Meta-Analysis of Retrospective Chart Reviews. Am Surg (2004) 70(7):593–9. 15279181

[9] Madani A van der Bilt JDW Consten ECJ Vriens MR Borel Rinkes IHM. Perforation in Appendiceal Well-Differentiated Carcinoid and Goblet Cell Tumors: Impact on Prognosis? A Systematic Review. Ann Surg Oncol (2015) 22(3):959–65. doi: 10.1245/s10434-014-4023-9 25190118

[10] Madsen AH Ladekarl M Villadsen GE Grønbæk H Sørensen MM Stribolt K. Effects of Cytoreductive Surgery and Hyperthermic Intraperitoneal Chemotherapy (HIPEC) in the Treatment of Goblet Cell Carcinoma: A Prospective Cohort Study. Ann Surg Oncol (2018) 25(2):422–30. doi: 10.1245/s10434-017-6272-x 29214450

[11] Minhas A Hendrickson J Minhas SA. Frequency and Risk Factors for Metastasis in Newly Diagnosed Appendiceal Carcinoma. Cureus (2021) 13(7): e16341. doi: 10.7759/cureus.16341 34395124 PMC8357020

[12] Wang G Li Q Chen W. Chemotherapy in the Treatment of Different Histological Types of Appendiceal Cancers: A SEER Based Study. BMC Cancer (2021) 21(1):778. doi: 10.1186/s12885-021-08502-3 34225672 PMC8259079

[13] Gibbs T Washington MK Eng C Idrees K Davis J Holowatyj AN. Histologic and Racial/Ethnic Patterns of Appendiceal Cancer Among Young Patients. Cancer Epidemiol Biomarkers Prev (2021) 30(6):1149–55. doi: 10.1158/1055-9965.EPI-20-1505 PMC8806661 33795212

[14] AlMasri S Nassour I Kowalsky SJ Hrebinko K Singhi AD Lee KK. The Role of Adjuvant Chemotherapy in Non-Metastatic Goblet Cell Carcinoid of the Appendix: An 11-Year Experience From the National Cancer Database. Ann Surg Oncol (2021) 28(7):3873–81. doi: 10.1245/s10434-020-09389-3 33231767

[15] Kowalsky SJ Nassour I AlMasri S Paniccia A Zureikat AH Choudry HA. Omission of Right Hemicolectomy May be Safe for Some Appendiceal Goblet Cell Adenocarcinomas: A Survival Analysis of the National Cancer Database. Ann Surg Oncol (2021) 28(13):8916–25. doi: 10.1245/s10434-021-10256-y 34409541

[16] Zheng M Li T Li Y Zhang T Zhang L Ma W. Survival Profile and Prognostic Factors for Appendiceal Mixed Neuroendocrine Non-Neuroendocrine Neoplasms: A SEER Population-Based Study. Front Oncol (2020) 10. doi: 10.3389/fonc.2020.01660 PMC7438709 32903647

[17] Mo S Zhou Z Ying Z Dai W Xiang W Han L. Epidemiology of and Prognostic Factors for Appendiceal Carcinomas: A Retrospective, Population-Based Study. Int J Colorectal Dis (2019) 34(11):1915–24. doi: 10.1007/s00384-019-03387-y 31642969

[18] Fields AC Lu P Enzinger A Goldberg J Irani J Bleday R. Treatment Patterns and Outcomes in Goblet Cell Carcinoid Tumors of the Appendix. J Surg Oncol (2019) 120(7):1096–101. doi: 10.1002/jso.25723 PMC7428861 31592538

[19] Onyemkpa C Davis A McLeod M Oyasiji T. Typical Carcinoids, Goblet Cell Carcinoids, Mixed Adenoneuroendocrine Carcinomas, Neuroendocrine Carcinomas and Adenocarcinomas of the Appendix: A Comparative Analysis of Survival Profile and Predictors. J Gastrointestinal Oncol (2019) 10(2):300–6. doi: 10.21037/jgo.2018.11.08 PMC6465489 31032098

[20] Brathwaite S Yearsley MM Bekaii-Saab T Wei L Schmidt CR Dillhoff ME. Appendiceal Mixed Adeno-Neuroendocrine Carcinoma: A Population-Based Study of the Surveillance, Epidemiology, and End Results Registry. Front Oncol (2016) 6. doi: 10.3389/fonc.2016.00148 PMC4904130 27379210

[21] Ayub A Parkash O Santana-Rodríguez N Raad W Bhora FY. Elevated Risk of Subsequent Malignancies in Patients With Appendiceal Cancer: A Population-Based Analysis. Indian J Gastroenterol (2016) 35(5):354–60. doi: 10.1007/s12664-016-0687-3 27595862

[22] Hsu C Rashid A Xing Y Chiang Y-J Chagpar RB Fournier KF. Varying Malignant Potential of Appendiceal Neuroendocrine Tumors: Importance of Histologic Subtype. J Surg Oncol (2013) 107(2):136–43. doi: 10.1002/jso.23205 22767417

[23] Turaga KK Pappas SG Gamblin TC. Importance of Histologic Subtype in the Staging of Appendiceal Tumors. Ann Surg Oncol (2012) 19(5):1379–85. doi: 10.1245/s10434-012-2238-1 22302267

[24] Landry CS. Analysis of 900 Appendiceal Carcinoid Tumors for a Proposed Predictive Staging System. Arch Surg (2008) 143(7):664–70. doi: 10.1001/archsurg.143.7.664 18645109

[25] McCusker ME Coté TR Clegg LX Sobin LH. Primary Malignant Neoplasms of the Appendix. Cancer (2002) 94(12):3307–12. doi: 10.1002/cncr.10589 12115365

[26] McGory ML Maggard MA Kang H O’Connell JB Ko CY. Malignancies of the Appendix: Beyond Case Series Reports. Dis Colon Rectum (2005) 48(12):2264–71. doi: 10.1007/s10350-005-0196-4 16258711

[27] Garach NR Kusamura S Guaglio M Bartolini V Deraco M Baratti D. Comparative Study of Mucinous and non-Mucinous Appendiceal Neoplasms With Peritoneal Dissemination Treated by Cyoreductive Surgery and Hyperthermic Intraperitoneal Chemotherapy (HIPEC). Eur J Surg Oncol (2021) 47(5):1132–39. doi: 10.1016/j.ejso.2020.08.017 33280949

[28] Alabraba E Pritchard DM Griffin R Diaz-Nieto R Banks M Cuthbertson DJ. Appendiceal Goblet Cell Carcinomas Have Poor Survival Despite Completion Surgery. Endocrine (2021) 73(3):734–44. doi: 10.1007/s12020-021-02727-9 33891259

[29] Barrak D Desale S Yoon JJ Dugan MM Kodavanti PP Sampah ME. Appendiceal Tumors With Glandular and Neuroendocrine Features Exhibiting Peritoneal Metastases - Critical Evaluation of Outcome Following Cytoreductive Surgery With Perioperative Chemotherapy. Eur J Surg Oncol (2021) 47(6):1278–85. doi: 10.1016/j.ejso.2021.01.010 33500181

[30] Berger Y Schuitevoerder D Vining CC Alpert L Fenton E Hindi E. Novel Application of Iterative Hyperthermic Intraperitoneal Chemotherapy for Unresectable Peritoneal Metastases From High-Grade Appendiceal Ex-Goblet Adenocarcinoma. Ann Surg Oncol (2021) 28(3):1777–85. doi: 10.1245/s10434-020-09064-7 32892267

[31] Zambrano-Vera K Sardi A Munoz-Zuluaga C Studeman K Nieroda C Sittig M. Outcomes in Peritoneal Carcinomatosis From Appendiceal Goblet Cell Carcinoma Treated With Cytoreductive Surgery and Hyperthermic Intraperitoneal Chemotherapy (CRS/HIPEC). Ann Surg Oncol (2020) 27(1):179–87. doi: 10.1245/s10434-019-07932-5 31646450

[32] McHugh KE Mukhopadhyay S Doxtader EE Lanigan C Allende DS. INSM1 Is a Highly Specific Marker of Neuroendocrine Differentiation in Primary Neoplasms of the Gastrointestinal Tract, Appendix, and Pancreas. Am J Clin Pathol (2020) 153(6):811–20. doi: 10.1093/ajcp/aqaa014 32128564

[33] Arai H Baca Y Battaglin F Kawanishi N Wang J Soni S. Molecular Characterization of Appendiceal Goblet Cell Carcinoid. Mol Cancer Ther (2020) 19(12):2634–40. doi: 10.1158/1535-7163.MCT-20-0318 PMC8011958 33037134

[34] Chai QD Pillai S Mcclure R Laycock A Wijesuriya R. Carcinoid Tumours of the Appendix: An Analysis of Emergency Appendicectomies Over a 24-Year Period and Outcomes of Laparoscopic Versus Open Resection. ANZ J Surg (2020) 90(10):1975–78. doi: 10.1111/ans.15879 32274843

[35] Munoz-Zuluaga CA King MC Diaz-Sarmiento VS Studeman K Sittig M MacDonald R. Defining “Complete Cytoreduction” After Cytoreductive Surgery and Hyperthermic Intraperitoneal Chemotherapy (CRS/HIPEC) for the Histopathologic Spectrum of Appendiceal Carcinomatosis. Ann Surg Oncol (2020) 27(13):5026–36. doi: 10.1245/s10434-020-08844-5 32705513

[36] Shyu S Choudry H Hall L Pingpank J Holtzman M Bartlett D. Clinicopathological Analysis of Appendiceal Goblet Cell Adenocarcinoma With Peritoneal Metastasis: World Health Organization Grade Predicts Survival Following Cytoreductive Surgery With Intraperitoneal Chemotherapy. Histopathology (2020) 77(5):798–809. doi: 10.1111/his.14189 32557796

[37] Das S Shi C Du L Idrees K Berlin J. Adenocarcinoma Ex-Goblet Cell: A Retrospective Experience. J Gastrointestinal Cancer (2019) 50(4):709–15. doi: 10.1007/s12029-018-0131-2 PMC6499718 29974346

[38] Yang C Gonzalez I Zhang L Cao D. Neuroendocrine Markers Insulinoma-Associated Protein 1, Chromogranin, Synaptophysin, and CD56 Show Rare Positivity in Adenocarcinoma Ex-Goblet Cell Carcinoids. Gastroenterol Res (2019) 12(3):120–7. doi: 10.14740/gr1138 PMC6575132 31236152

[39] Clift AK Kornasiewicz O Drymousis P Faiz O Wasan HS Kinross JM. Goblet Cell Carcinomas of the Appendix: Rare But Aggressive Neoplasms With Challenging Management. Endocrine Connections (2018) 7(2):268–77. doi: 10.1530/EC-17-0311 PMC5801558 29335251

[40] Yozu M Johncilla ME Srivastava A Ryan DP Cusack JC Doyle L. Histologic and Outcome Study Supports Reclassifying Appendiceal Goblet Cell Carcinoids as Goblet Cell Adenocarcinomas, and Grading and Staging Similarly to Colonic Adenocarcinomas. Am J Surg Pathol (2018) 42(7):898–910. doi: 10.1097/PAS.0000000000001056 29579011

[41] Wen KW Grenert JP Joseph NM Shafizadeh N Huang A Hosseini M. Genomic Profile of Appendiceal Goblet Cell Carcinoid is Distinct Compared to Appendiceal Neuroendocrine Tumor and Conventional Adenocarcinoma. Hum Pathol (2018) 77:166–74. doi: 10.1016/j.humpath.2018.03.026 29634977

[42] Jesinghaus M Konukiewitz B Foersch S Stenzinger A Steiger K Muckenhuber A. Appendiceal Goblet Cell Carcinoids and Adenocarcinomas Ex-Goblet Cell Carcinoid are Genetically Distinct From Primary Colorectal-Type Adenocarcinoma of the Appendix. Modern Pathol (2018) 31(5)829–9. doi: 10.1038/modpathol.2017.184 29327707

[43] Şenel F Karaman H Demir H. Neuroendocrine Tumors Detected in Appendectomy Specimens: Ten-Year Single-Center Experience. Turkish J Med Sci (2018) 48:68–73. doi: 10.3906/sag-1709-37 29479957

[44] Nonaka D Papaxoinis G Lamarca A Fulford P Valle J Chakrabarty B. A Study of Appendiceal Crypt Cell Adenocarcinoma (So-Called Goblet Cell Carcinoid and its Related Adenocarcinoma). Hum Pathol (2018) 72:18–27. doi: 10.1016/j.humpath.2017.08.005 28823572

[45] Johncilla M Stachler M Misdraji J Lisovsky M Yozu M Lindeman N. Mutational Landscape of Goblet Cell Carcinoids and Adenocarcinoma Ex Goblet Cell Carcinoids of the Appendix is Distinct From Typical Carcinoids and Colorectal Adenocarcinomas. Modern Pathol (2018) 31(6):989–96. doi: 10.1038/s41379-018-0003-0 29422640

[46] Yang C Sun L Zhang L Zhou L Niu D Cao W. SATB2 Shows Different Profiles Between Appendiceal Adenocarcinomas Ex Goblet Cell Carcinoids and Appendiceal/Colorectal Conventional Adenocarcinomas: An Immunohistochemical Study With Comparison to CDX2. Gastroenterol Res (2018) 11(3):221–30. doi: 10.14740/gr1015w PMC5997472 29915633

[47] Yu H-H Yonemura Y Hsieh M-C Mizumoto A Wakama S Lu C-Y. Cytoreductive Surgery and Hyperthermic Intraperitoneal Chemotherapy for Appendiceal Goblet Cell Carcinomas With Peritoneal Carcinomatosis: Results From a Single Specialized Center. Cancer Manage Res (2017) 9:513–23. doi: 10.2147/CMAR.S147227 PMC5655158 29089784

[48] Gui X Meng Z McConnell YJ Liu S Falck VG Mack LA. Differing Expression Profiles of Notch /Enterocyte and Wnt /Secretory Lineage Signallings are Associated With Morphological Diversity of Appendiceal Tumours. J Clin Pathol (2017) 70(1):40–50. doi: 10.1136/jclinpath-2016-203645 27371613

[49] Reid MD Basturk O Shaib WL Xue Y Balci S Choi H-J. Adenocarcinoma Ex-Goblet Cell Carcinoid (Appendiceal-Type Crypt Cell Adenocarcinoma) is a Morphologically Distinct Entity With Highly Aggressive Behavior and Frequent Association With Peritoneal/Intra-Abdominal Dissemination: An Analysis of 77 Cases. Modern Pathol (2016) 29(10):1243–53. doi: 10.1038/modpathol.2016.105 PMC5389379 27338636

[50] Ihemelandu C Sugarbaker PH. Clinicopathologic and Prognostic Features in Patients With Peritoneal Metastasis From Mucinous Adenocarcinoma, Adenocarcinoma With Signet Ring Cells, and Adenocarcinoid of the Appendix Treated With Cytoreductive Surgery and Perioperative Intraperitoneal Chemotherapy. Ann Surg Oncol (2016) 23(5):1474–80. doi: 10.1245/s10434-015-4995-0 26597367

[51] Radomski M Pai RK Shuai Y Ramalingam L Jones H Holtzman MP. Curative Surgical Resection as a Component of Multimodality Therapy for Peritoneal Metastases From Goblet Cell Carcinoids. Ann Surg Oncol (2016) 23(13):4338–43. doi: 10.1245/s10434-016-5412-z 27401448

[52] Lamarca A Nonaka D Lopez Escola C Hubner RA O’Dwyer S Chakrabarty B. Appendiceal Goblet Cell Carcinoids: Management Considerations From a Reference Peritoneal Tumour Service Centre and ENETS Centre of Excellence. Neuroendocrinology (2016) 103(5):500–17. doi: 10.1159/000440725 26356507

[53] Brathwaite S Rock J Yearsley MM Bekaii-Saab T Wei L Frankel WL. Mixed Adeno-Neuroendocrine Carcinoma: An Aggressive Clinical Entity. Ann Surg Oncol (2016) 23(7):2281–86. doi: 10.1245/s10434-016-5179-2 PMC4891253 26965701

[54] Rossi RE Luong T-V Caplin ME Thirlwell C Meyer T Garcia-Hernandez J. Goblet Cell Appendiceal Tumors – Management Dilemmas and Long-Term Outcomes. Surg Oncol (2015) 24(1):47–53. doi: 10.1016/j.suronc.2015.01.001 25686643

[55] Taggart MW Abraham SC Overman MJ Mansfield PF Rashid A. Goblet Cell Carcinoid Tumor, Mixed Goblet Cell Carcinoid-Adenocarcinoma, and Adenocarcinoma of the Appendix: Comparison of Clinicopathologic Features and Prognosis. Arch Pathol Lab Med (2015) 139(6):782–90. doi: 10.5858/arpa.2013-0047-OA 26030247

[56] Nash GM Smith JD Tang L Weiser MR Temple LK O’Reilly E. Lymph Node Metastasis Predicts Disease Recurrence in a Single-Center Experience of 70 Stages 1–3 Appendix Cancers: A Retrospective Review. Ann Surg Oncol (2015) 22(11):3613–17. doi: 10.1245/s10434-015-4415-5 PMC4878821 25663593

[57] Randle RW Griffith KF Fino NF Swett KR Stewart JH Shen P. Appendiceal Goblet Cell Carcinomatosis Treated With Cytoreductive Surgery and Hyperthermic Intraperitoneal Chemotherapy. J Surg Res (2015) 196(2):229–34. doi: 10.1016/j.jss.2015.03.051 PMC4514020 25881787

[58] Olsen IH Holt N Langer SW Hasselby JP Grønbæk H Hillingsø J. Goblet Cell Carcinoids: Characteristics of a Danish Cohort of 83 Patients. PloS One (2015) 10(2):e0117627. doi: 10.1371/journal.pone.0117627 25671432 PMC4324995

[59] Dimmler A Geddert H Faller G. EGFR, KRAS, BRAF-Mutations and Microsatellite Instability are Absent in Goblet Cell Carcinoids of the Appendix. Pathol Res Practice (2014) 210(5):274–8. doi: 10.1016/j.prp.2014.01.002 24560515

[60] McConnell YJ Mack LA Gui X Carr NJ Sideris L Temple WJ. Cytoreductive Surgery With Hyperthermic Intraperitoneal Chemotherapy: An Emerging Treatment Option for Advanced Goblet Cell Tumors of the Appendix. Ann Surg Oncol (2014) 21(6):1975–82. doi: 10.1245/s10434-013-3469-5 24398544

[61] Ng D Falck V McConnell YJ Mack LA Temple WJ Gui X. Appendiceal Goblet Cell Carcinoid and Mucinous Neoplasms are Closely Associated Tumors: Lessons From Their Coexistence in Primary Tumors and Concurrence in Peritoneal Dissemination. J Surg Oncol (2014) 109(6):548–55. doi: 10.1002/jso.23537 24374723

[62] Lee KS Tang LH Shia J Paty PB Weiser MR Guillem JG. Goblet Cell Carcinoid Neoplasm of the Appendix: Clinical and CT Features. Eur J Radiol (2013) 82(1):85–89. doi: 10.1016/j.ejrad.2012.05.038 23088880

[63] Jiang Y Long H Li T Wang W Liu H Zhang X. Schistosomiasis May Contribute To Goblet Cell Carcinoid of the Appendix. J Parasitol (2012) 98(3):565–68. doi: 10.1645/JP-GE-2865.1 22746391

[64] Liu E Telem DA Warner RRP Dikman A Divino CM. The Role of Ki-67 in Predicting Biological Behavior of Goblet Cell Carcinoid Tumor in Appendix. Am J Surg (2011) 202(4):400–3. doi: 10.1016/j.amjsurg.2010.08.036 21824598

[65] Cashin P Nygren P Hellman P Granberg D Andréasson H Mahteme H. Appendiceal Adenocarcinoids With Peritoneal Carcinomatosis Treated With Cytoreductive Surgery and Intraperitoneal Chemotherapy: A Retrospective Study of *In Vitro* Drug Sensitivity and Survival. Clin Colorectal Cancer (2011) 10(2):108–12. doi: 10.1016/j.clcc.2011.03.006 21859562

[66] Jiang Y Liu H Long H Yang Y Liao D Zhang X. Goblet Cell Carcinoid of the Appendix: A Clinicopathological and Immunohistochemical Study of 26 Cases From Southwest China. Int J Surg Pathol (2010) 18(6):488–92. doi: 10.1177/1066896910379404 20732910

[67] Yan TD Brun EA Sugarbaker PH. Discordant Histology of Primary Appendiceal Adenocarcinoid Neoplasms With Peritoneal Dissemination. Ann Surg Oncol (2008) 15(5):1440–46. doi: 10.1245/s10434-007-9754-4 18299936

[68] van Eeden S Offerhaus GJA Hart AAM Boerrigter L Nederlof PM Porter E. Goblet Cell Carcinoid of the Appendix: A Specific Type of Carcinoma. Histopathology (2007) 51(6):763–73. doi: 10.1111/j.1365-2559.2007.02883.x 18042066

[69] Alsaad KO Serra S Schmitt A Perren A Chetty R. Cytokeratins 7 and 20 Immunoexpression Profile in Goblet Cell and Classical Carcinoids of Appendix. Endocrine Pathol (2007) 18(1):16–22. doi: 10.1007/s12022-007-0004-x 17652796

[70] Alsaad KO Serra S Perren A Hsieh E Chetty R. CK19 and CD99 Immunoexpression Profile in Goblet Cell (Mucin-Producing Neuroendocrine Tumors) and Classical Carcinoids of the Vermiform Appendix. Int J Surg Pathol (2007) 15(3):252–7. doi: 10.1177/1066896907302118 17652531

[71] Byrn JC Wang J-L Divino CM Nguyen SQ Warner RRP. Management of Goblet Cell Carcinoid. J Surg Oncol (2006) 94(5):396–402. doi: 10.1002/jso.20587 16967437

[72] Modlin IM Kidd M Latich I Zikusoka MN Eick GN Mane SM. Genetic Differentiation of Appendiceal Tumor Malignancy. Ann Surg (2006) 244(1):52–60. doi: 10.1097/01.sla.0000217617.06782.d5 16794389 PMC1570599

[73] Pham TH Wolff B Abraham SC Drelichman E. Surgical and Chemotherapy Treatment Outcomes of Goblet Cell Carcinoid: A Tertiary Cancer Center Experience. Ann Surg Oncol (2006) 13(3):370–6. doi: 10.1245/ASO.2006.02.016 16485156

[74] Mahteme H Sugarbaker PH. Treatment of Peritoneal Carcinomatosis From Adenocarcinoid of Appendiceal Origin. Br J Surg (2004) 91(9):1168–73. doi: 10.1002/bjs.4609 15449269

[75] Stancu M. Genetic Alterations in Goblet Cell Carcinoids of the Vermiform Appendix and Comparison With Gastrointestinal Carcinoid Tumors. Modern Pathol (2003) 16(12):1189–98. doi: 10.1097/01.MP.0000097362.10330.B1 14681318

[76] Kende AI Carr NJ Sobin LH. Expression of Cytokeratins 7 and 20 in Carcinomas of the Gastrointestinal Tract. Histopathology (2003) 42(2):137–140. doi: 10.1046/j.1365-2559.2003.01545.x 12558745

[77] Li CC Hirokawa M Qian ZR Xu B Sano T. Expression of E-Cadherin, β-Catenin, and Ki-67 in Goblet Cell Carcinoids of the Appendix: An Immunohistochemical Study With Clinical Correlation. Endocrine Pathol (2002) 13(1):47–58. doi: 10.1385/EP:13:1:47 12114750

[78] Kanthan R Saxena A Kanthan SC. Goblet Cell Carcinoids of the Appendix. Arch Pathol Lab Med (2001) 125(3):386–90. doi: 10.5858/2001-125-0386-GCCOTA 11231488

[79] Ramnani DM Wistuba II Behrens C Gazdar AF Sobin LH Albores-Saavedra J. K-Ras and P53 Mutations in the Pathogenesis of Classical and Goblet Cell Carcinoids of the Appendix. Cancer (1999) 86(1):14–21. doi: 10.1002/(SICI)1097-0142(19990701)86:1<14::AID-CNCR4>3.0.CO;2-X 10391558

[80] Anderson NH Somerville JE Johnston CF Hayes DM Buchanan KD Sloan JM. Appendiceal Goblet Cell Carcinoids: A Clinicopathological and Immunohistochemical Study. Histopathology (1991) 18(1):61–65. doi: 10.1111/j.1365-2559.1991.tb00815.x 1672861

[81] Park K Blessing K Kerr K Chetty U Gilmour H. Goblet Cell Carcinoid of the Appendix. Gut (1990) 31(3):840–840. doi: 10.1136/gut.31.3.322 PMC1378275 2323597

[82] Burke AP Sobin LH Federspiel BH Shekitka KM Helwig EB. Goblet Cell Carcinoids and Related Tumors of the Vermiform Appendix. Am J Clin Pathol (1990) 94(1):27–35. doi: 10.1093/ajcp/94.1.27 2163192

[83] Bak M Asschenfeldt P. Adenocarcinoid of the Vermiform Appendix. Dis Colon Rectum (1988) 31(8):605–12. doi: 10.1007/BF02556796 3402286

[84] Watson PH Alguacil-Garcia A. Mixed Crypt Cell Carcinoma. Virchows Archiv A Pathol Anat Histopathol (1987) 412(2):175–82. doi: 10.1007/BF00716191 3122418

[85] Edmonds P Merino MJ LiVolsi VA Duray PH. Adenocarcinoid (Mucinous Carcinoid) of the Appendix. Gastroenterology (1984) 86(2):302–9. doi: 10.1016/0016-5085(84)90415-3 6690357

[86] Höfler H Klöppel G Heitz PU. Combined Production of Mucus, Amines and Peptides by Goblet-Cell Carcinoids of the Appendix and Ileum. Pathol Res Practice (1984) 178(6):555–61. doi: 10.1016/S0344-0338(84)80088-6 6483683

[87] Isaacson P. Crypt Cell Carcinoma of the Appendix (So-Called Adenocarcinoid Tumor). Am J Surg Pathol (1981) 5(3):213–24. doi: 10.1097/00000478-198104000-00001 7235117

[88] Warkel RL Cooper PH Helwig EB. Adenocarcinoid, a Mucin-Producing Carcinoid Tumor of the Appendix. A Study of 39 Cases. Cancer (1978) 42(6):2781–93. doi: 10.1002/1097-0142(197812)42:6<2781::aid-cncr2820420638>3.0.co;2-b 728874

[89] Tsang ES McConnell YJ Schaeffer DF Lee L Yin Y Zerhouni S. Outcomes of Surgical and Chemotherapeutic Treatments of Goblet Cell Carcinoid Tumors of the Appendix. Ann Surg Oncol (2018) 25(8):2391–99. doi: 10.1245/s10434-018-6560-0 29916007

[90] Lee LH McConnell YJ Tsang E Zerhouni S Speers C Kennecke H. Simplified 2-Tier Histologic Grading System Accurately Predicts Outcomes in Goblet Cell Carcinoid of the Appendix. Hum Pathol (2015), 46(12). doi: 10.1016/j.humpath.2015.08.005 26433702

[91] Jedrzkiewicz J Tateishi Y Kirsch R Conner J Bischof D McCart A. Impact of Referral Center Pathology Review on Diagnosis and Management of Patients With Appendiceal Neoplasms. Arch Pathol Lab Med (2020) 144(6):764–68. doi: 10.5858/arpa.2019-0214-OA 31714810

[92] Arnold CA Graham RP Jain D Kakar S Lam-Himlin DM Naini BV. Knowledge Gaps in the Appendix: A Multi-Institutional Study From Seven Academic Centers. Modern Pathol (2019) 32(7):988–96:. doi: 10.1038/s41379-019-0216-x 30765881

[93] Maedler C Arnason T Dorreen A Sapp H Castonguay M Murphy J. Goblet Cell Carcinoid of the Appendix – An Interobserver Variability Study Using Two Proposed Classification Systems. Ann Diagn Pathol (2018) 32:51–5. doi: 10.1016/j.anndiagpath.2017.11.001 29414399

[94] Wen KW Hale G Shafizadeh N Hosseini M Huang A Kakar S. Appendiceal Goblet Cell Carcinoid: Common Errors in Staging and Clinical Interpretation With a Proposal for an Improved Terminology. Hum Pathol (2017) 65. doi: 10.1016/j.humpath.2017.05.012 28551326

[95] Wang Y Shahabi A Loeffler A. Appendiceal Goblet Cell Adenocarcinoma. Arch Pathol Lab Med (2022). doi: 10.5858/arpa.2021-0249-RA 35142802

[96] Boyajian H Majeski V Flores A Sturtz D Baidoun F Dughayli M. Clinicopathological and Perioperative Outcome of Appendiceal Tumors: Case Review of 31 Patients. Spartan Med Res J (2020) 5(2):13487. doi: 10.51894/001c.13487 33655185 PMC7746073

[97] Jimenez DS Mentzer CJ Mount MG Orr RK Thurston BC. Goblet Cell Carcinoid Tumors During Emergent General Surgery. Am Surgeon (2020) 86(11):1584–5. doi: 10.1177/0003134820940263 32841045

[98] Prieto-Nieto MI Pastor D Rodríguez-Cobos J Pérez JP Méndez C Palacios E. Δnp73 Status in Peritoneal and Ovarian Dissemination of Appendicular Adenocarcinoids (Goblet Cells). Clin Trans Oncol (2019) 21(10):1432–9. doi: 10.1007/s12094-019-02091-1 31025168

[99] Karaman H Şenel F Güreli M Ekinci T Topuz Ö. Goblet Cell Carcinoid of the Appendix and Mixed Adenoneuroendocrine Carcinoma: Report of Three Cases. World J Gastrointestinal Oncol (2017) 9(7):308–13. doi: 10.4251/wjgo.v9.i7.308 PMC5534399 28808504

[100] Macak J Nemejcova K Dvorackova J. Are Goblet Cell Carcinoids a Group of Heterogeneous Tumors? Biomed Papers (2017) 161(3):281–85. doi: 10.5507/bp.2017.027 28659644

[101] Piao J Veerapong J. Adenocarcinoma Ex Goblet Cell Carcinoid (GCC) of the Appendix: Report of Five Cases and Pitfalls in Diagnosis of GCC. Arch Surg Oncol (2016) 02(01):108. doi: 10.4172/2471-2671.1000108

[102] Toumpanakis C Standish RA Baishnab E Winslet MC Caplin ME. Goblet Cell Carcinoid Tumors (Adenocarcinoid) of the Appendix. Dis Colon Rectum (2007) 50(3):315–22. doi: 10.1007/s10350-006-0762-4 17195086

[103] Bucher P Gervaz P Ris F Oulhaci W Egger J-F Morel P. Surgical Treatment of Appendiceal Adenocarcinoid (Goblet Cell Carcinoid). World J Surg (2005) 29(11):1436–39. doi: 10.1007/s00268-005-7958-y 16136284

[104] Lin BT Gown AM. Mixed Carcinoid and Adenocarcinoma of the Appendix. Appl Immunohistochem Mol Morphol (2004) 12(3):271–6. doi: 10.1097/00129039-200409000-00015 15551743

[105] Butler JA Houshiar A Lin F Wilson SE. Goblet Cell Carcinoid of the Appendix. Am J Surg (1994) 168(6):685–7. doi: 10.1016/S0002-9610(05)80145-X 7978019

[106] Olsson B Ljungberg O. Adenocarcinoid of the Vermiform Appendix. Virchows Archiv A Pathol Anat Histol (1980) 386(2):201–10. doi: 10.1007/BF00427232 7368563

[107] Chen V Qizilbash AH. Goblet Cell Carcinoid Tumor of the Appendix. Report of Five Cases and Review of the Literature. Arch Pathol Lab Med (1979) 103(4):180–2. 218519

[108] Hallet J Law CHL Cukier M Saskin R Liu N Singh S. Exploring the Rising Incidence of Neuroendocrine Tumors: A Population-Based Analysis of Epidemiology, Metastatic Presentation, and Outcomes. Cancer (2015) 121(4):589–97. doi: 10.1002/cncr.29099 25312765

[109] Pape U-F Perren A Niederle B Gross D Gress T Costa F. ENETS Consensus Guidelines for the Management of Patients With Neuroendocrine Neoplasms From the Jejuno-Ileum and the Appendix Including Goblet Cell Carcinomas. Neuroendocrinology (2012) 95(2):135–56. doi: 10.1159/000335629 22262080

[110] Plöckinger U Couvelard A Falconi M Sundin A Salazar R Christ E. Consensus Guidelines for the Management of Patients With Digestive Neuroendocrine Tumours: Well-Differentiated Tumour/Carcinoma of the Appendix and Goblet Cell Carcinoma. Neuroendocrinology (2008) 87(1):20–30. doi: 10.1159/000109876 17934252

[111] Nagtegaal ID Odze RD Klimstra D Paradis V Rugge M Schirmacher P. The 2019 WHO Classification of Tumours of the Digestive System. Histopathology (2020) 76(2):182–8. doi: 10.1111/his.13975 PMC7003895 31433515

[112] Office for National Statistics. 2011 Census: Aggregate Data. (London) (2011)

[113] UK Government. The English Indices of Deprivation 2019 (2019). Available at: https://assets.publishing.service.gov.uk/government/uploads/system/uploads/attachment_data/file/853811/IoD2019_FAQ_v4.pdf.

[114] Abdel-Rahman O Fazio N. Sex-Based Differences in Prognosis of Patients With Gastroenteropancreatic-Neuroendocrine Neoplasms. Pancreas (2021) 50(5):727–31. doi: 10.1097/MPA.0000000000001821 34016894

